# Retraction Notice to: Silencing of Long Non-coding RNA HOTTIP Reduces Inflammation in Rheumatoid Arthritis by Demethylation of SFRP1

**DOI:** 10.1016/j.omtn.2022.05.014

**Published:** 2022-05-13

**Authors:** Xumin Hu, Jianhua Tang, Xuyun Hu, Peng Bao, Weixi Deng, Jionglin Wu, Yuwei Liang, Zhipeng Chen, Liangbin Gao, Yong Tang

## Main Text

(Molecular Therapy: Nucleic Acids *19*, 468–481; March 2020)

This article has been retracted at the request of the editors of *Molecular Therapy – Nucleic Acids*.

After concerns were raised by a reader, evidence for image duplication in identical or altered fashion in Figures 5B and 5C were identified. This reuse (and in part, misrepresentation) of data without appropriate attribution represents a severe abuse of the scientific publishing system. The authors have agreed to the retraction.

